# Clinic Predictive Factors for Insufficient Myocardial Reperfusion in ST-Segment Elevation Myocardial Infarction Patients Treated with Selective Aspiration Thrombectomy during Primary Percutaneous Coronary Intervention

**DOI:** 10.1155/2016/3823809

**Published:** 2016-11-07

**Authors:** Jinfan Tian, Yue Liu, Xiantao Song, Min Zhang, Feng Xu, Fei Yuan, Shuzheng Lyu

**Affiliations:** ^1^Department of Cardiology, Beijing Anzhen Hospital, Capital Medical University, Beijing, China; ^2^Beijing Institute of Heart, Lung and Blood Vessel Diseases, Beijing, China; ^3^Cardiovascular Disease Centre of Xiyuan Hospital, China Academy of Chinese Medical Sciences, Beijing, China

## Abstract

*Background*. Insufficient data are available on the potential benefit of selective aspiration and clinical predictors for no-reflow in STEMI patients undergoing primary percutaneous coronary intervention (PPCI) adjunct with aspiration thrombectomy.* Objective*. The aim of our study was to investigate clinical predictors for insufficient reperfusion in patients with high thrombus burden treated with PPCI and manual aspiration thrombectomy.* Methods*. From January 2011 till December 2015, 277 STEMI patients undergoing manual aspiration thrombectomy and PPCI were selected and 202 patients with a Thrombolysis in Myocardial Infarction (TIMI) thrombus grade 4~5 were eventually involved in our study. According to a cTFC value, patients were divided into Group I (cTFC > 40), namely, insufficient reperfusion group; Group II (cTFC ≤ 40), namely, sufficient reperfusion group.* Results*. Univariate analysis showed that hypertension, multivessel disease, time from symptom to PCI (≧4.8 hours), and postaspiration cTFC > 40 were negative predictors for insufficient reperfusion. After multivariate adjustment, age ≧ 60 years, hypertension, time from symptom to PCI (≧4.8 hours), and postaspiration cTFC > 40 were independently associated with insufficient reperfusion in STEMI patients treated with manual aspiration thrombectomy. Upfront intracoronary GP IIb/IIIa inhibitor (Tirofiban) was positively associated with improved myocardial reperfusion.* Conclusion*. Fully identifying risk factors will help to improve the effectiveness of selective thrombus aspiration.

## 1. Introduction

The common pathophysiological mechanism of acute coronary syndrome is sudden disruption of coronary arterial plaque due to rupture, fissuring, or superficial erosion leading to obstructive intracoronary thrombosis [1]. Acute ST-segment elevation myocardial infarction (STEMI) is characterized by complete thrombotic occlusion of a coronary artery [2]. Currently, primary percutaneous coronary intervention (PPCI) is the most effective treatment of STEMI. However, restoring the epicardial coronary is not always translated into optimum myocardial reperfusion, and this is a phenomenon at microvascular level called no-reflow [3]. No-reflow is associated with mortality and other major adverse events [4]. The pathogenesis of no-reflow is multifactorial, including ischemic damage, reperfusion injury, distal embolization, and individual susceptibility [5]. Previous studies have found that higher thrombus burden in patients with STEMI is independently associated with distal embolization, no-reflow phenomenon, major adverse cardiac events, sent thrombosis, and death [6, 7]. Removal of the thrombus by manual thrombectomy before sent deployment has the potential of reducing distal embolization and improving microvascular perfusion [8]. The TAPAS (Thrombus Aspiration during Percutaneous Coronary Intervention in Acute Myocardial Infarction Study) showed improved myocardial blush grade (MBG) and better clinical outcomes with the thrombus aspiration than conventional PCI [9]. However, the TASTE trial (Thrombus Aspiration in STEMI in Scandinavia) failed to show a significant benefit of routine thrombectomy in terms of early and medium-term mortality [10, 11]. In addition, the most recent and so far the largest trial showed that routine manual thrombectomy did not reduce the primary composite endpoint of cardiovascular death, recurrent MI, cardiogenic shock, or New York Heart Association class IV heart failure at 180 days, or the individual components of the primary endpoint, stent thrombosis, or target-vessel revascularization. Rates of TIMI 3 flow after PCI were the same in routine manual thrombectomy group and PCI alone treatment group and were similar for no-reflow rates on angiography. Subgroup patients with high thrombus burden and initial TIMI flow grade 0~1 also showed no benefits [12]. On the basis of the results of these studies, the prior class IIa recommendation for aspiration thrombectomy has been changed. Routine aspiration thrombectomy during primary PCI is now not recommended. Why these randomized trials showed limited clinical benefit of routine aspiration remains elusive. Furthermore, there are insufficient data to assess the potential benefit of a strategy of selective aspiration thrombectomy and to show who might benefit most from selective aspiration. It has been observed in a real world that patients with TIMI thrombus grade 4~5 or with initial TIMI flow 0~1 are more often treated with thrombus aspiration. Detecting of clinical predictors for insufficient myocardial reperfusion during PPCI will help patients with high thrombus burden to benefit most from thrombus aspiration, consequently improving the benefit of manual thrombus aspiration in these patients.

## 2. Methods

### 2.1. Patient Population

277 STEMI patients undergoing successful primary from January 2011 to December 2015 in Beijing Anzhen Hospital were initially retrospectively recruited in our study, and thrombus aspiration was performed before PPCI on all the patients. 202 patients with TIMI thrombus burden grade 4~5 were eventually included in our study. STEMI was defined as (1) typical ischemic chest pain with a duration time of more than 30 minutes (the pain could not be alleviated by resting or taking nitroglycerin); (2) ST-segment elevation >1 mm in two consecutive leads or the onset of left bundle branch block; (3) detection of rise and/or fall of cardiac biomarker values (preferably troponin) with at least one value above the 99th percentile of the upper reference limit. Patients who presented with non-STEMI or had undergone a coronary artery bypass surgery were excluded.

### 2.2. Angiographic Analysis

All patients received 300 mg chewable aspirin and a 300 mg loading dose of clopidogrel on admission and 50–70 U/kg intravenous standard heparin before the procedure. Coronary angiography was performed according to the standard criteria. The thrombectomy was determined by the experienced cardiologist commonly according to the visual assessments for thrombus. The administration of glycoprotein IIb/IIIa receptor inhibitor (Tirofiban) was left to the operator's discretion during PCI. The blood flow in the infarct-related artery (IRA) was graded according to the Thrombolysis in Myocardial Infarction (TIMI) grading system. Corrected TIMI frame counts (cTFC) values, the degree of stenosis, and TIMI thrombus burden grade were measured by quantitative coronary arteriography offline by two experienced interventional cardiologists who were blinded to the clinical data of the patients. The coronary angiograms were acquired at 15 frames/second with a digital angiographic system. Data were then converted to the most common filming speed of 30 frames/second by multiplying by a factor of two. cTFC was regarded as 100 for flows not reaching the distal reference point.

Insufficient reperfusion was diagnosed with a cTFC value > 40 in the IRA. A value ≤ 40 was taken to indicate sufficient reperfusion [13, 14]. Accordingly, the patients were subdivided into Group I (those with insufficient reperfusion) and Group II (those with sufficient reperfusion). Thrombus burden was scored in five degrees: G0 = no thrombus, G1 = possible thrombus, G2 = small [greatest dimension ≤ 1/2 vessel diameter (VD)], G3 = moderate (>1/2 but < 2VD), G4 = large (≥ 2VD), and G5 = total occlusion. TIMI thrombus score ≧ 4 was defined as high-grade angiographic thrombus burden [15]. After the intervention, all patients were given clopidogrel 75 mg and aspirin 100 mg once daily for 12 months. Other routine medications were according to the guideline recently.

### 2.3. Data Collected

Variables including demographics, medical history, laboratory studies, and procedural characteristics were recorded by two independent researchers who were blinded to the study objectives.

### 2.4. Statistical Analysis

Continuous variables are expressed as mean ± standard deviation. Normal distribution continuous variables were compared using Student's* t*-test, while Mann–Whitney* U* test was used for abnormal distribution. Categorical variables are expressed as number and percentage of patients. The chi-square test or Fish's exact test was used to analyze categorical variables. Univariate and multivariate analysis were performed to identify independent predictors of insufficient reperfusion phenomenon. In multivariable models, covariates included those with a* P* value < 0.1 in the univariable analysis and those that were clinically relevant. Results were presented as adjusted OR with 95% CI. Any correlation between the data was tested by the Pearson correlation analysis. The receiver operating characteristic (ROC) was used to determine the relationship between time from symptom to PCI and post-PCI cTFC and to determine the correlation between postaspiration cTFC and post-PCI cTFC. Cut-off level of time from symptom to PCI was also calculated by ROC curve. Statistical analysis was made using SPSS 17.0. A* P* value < 0.05 was considered statistically significant.

## 3. Results

### 3.1. Baseline Clinical Characteristics

The study population consisted of 202 STEMI patients (age 56 ± 11 years, male 87.6%) with high thrombus burden undergoing manual thrombus aspiration and PPCI. The incidence of insufficient reperfusion was 15.3% (*n* = 31). Compared to Group II, there were more patients older than 60 years in Group I (51.6% versus 33.3%), but the difference was statistically different. In Group I, 77.4% of the patients were with hypertension, while 47.4% of the patients were with hypertension in Group II. In respect to gender, smoking, diabetes, and history of PCI, there were no significant differences between Group I and Group II. In respect to SBP, DBP, Killip classification, and laboratory findings, the differences were not statistically different between the two groups (see Table 1).

### 3.2. Percutaneous Intervention Findings

**Figure 1:**
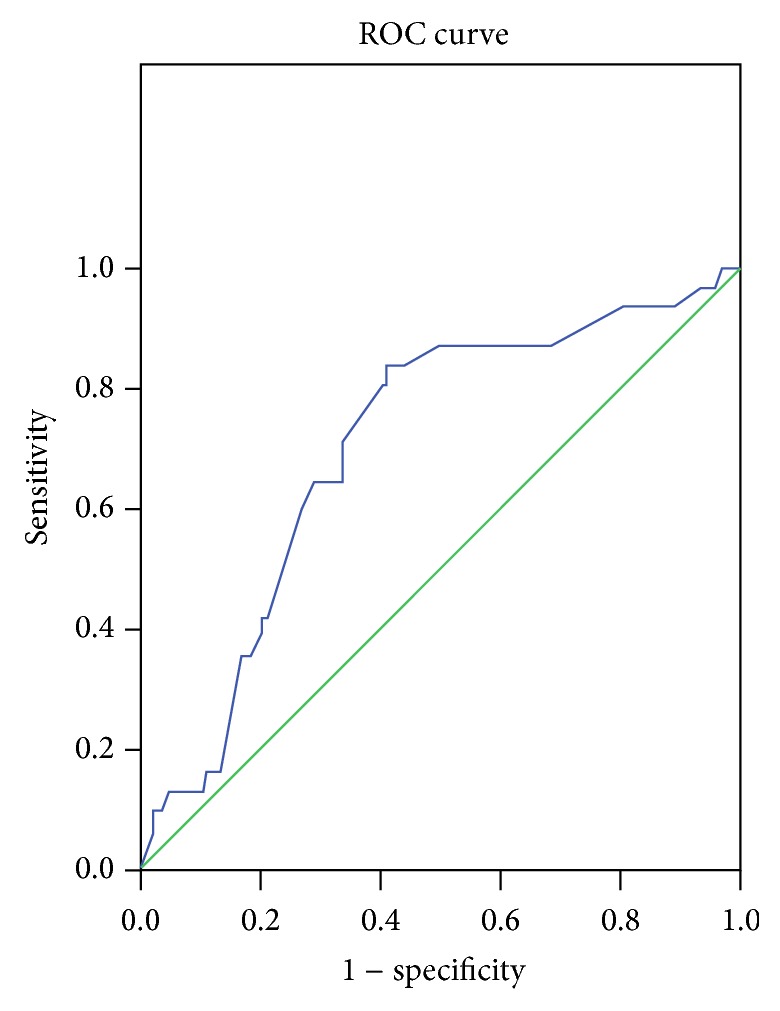
ROC analysis of time from symptom to PCI for insufficient reperfusion (AUC 0.701, 95% CI: 0.605–0.797, *P* = 0.000).

Compared to Group II, Group I has a longer time from symptom to PCI, and the difference was statistically significant. The ROC curve was calculated for time from symptom and was illustrated in Figure 1. The area under the curve was 0.701 and showed a significant *P* value. The cut-off level of time from symptom was 4.8 hours with 83.9% sensitivity and 57.1% specificity. Compared with Group II, there were more patients with multivessel disease in Group I with a significant* P* value. The cTFC postaspiration in Group I was significantly higher than that in Group II, and there were more patients with postaspiration cTFC > 40 in Group I. Compared to Group II, there were more patients with postaspiration TIMI flow grade 0 in Group I. There were no differences between the two groups in respect to initial cTFC value and initial degree of luminal stenosis. In respect to infarct-related artery, infarct location, and number of stents, no statistical differences were found between the two groups. There was also no difference between the two groups according to upfront intracoronary or intravenous administration of GP IIb/IIIa inhibitors (see Table 2).

### 3.3. Correlation between Post-PCI cTFC and Other Parameters

Correlation between post-PCI cTFC and other parameters measured by QCA was illustrated in Table 3. Significant positive correlation was demonstrated between postaspiration cTFC and post-PCI cTFC (*r* = 0.569, *P* = 0.000). There was no significant correlation between post-PCI cTFC and initial cTFC (*r* = 0.015, *P* = 0.832).

**Figure 2:**
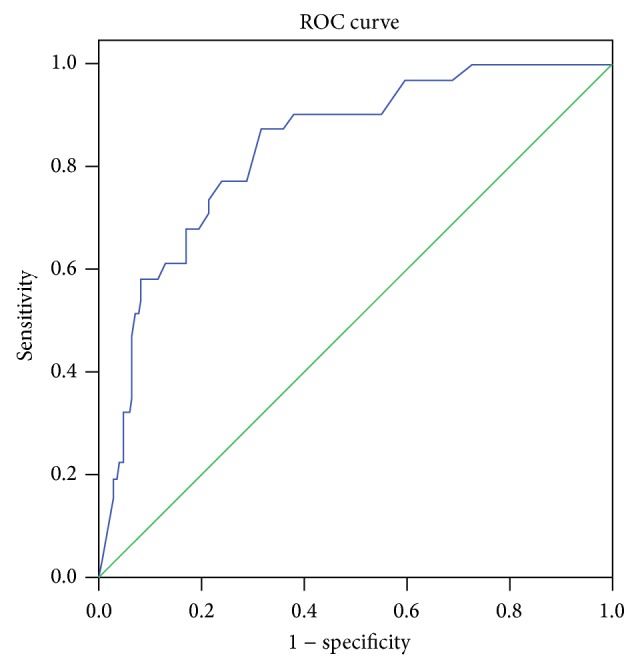
ROC analysis of postaspiration cTFC for insufficient reperfusion (AUC 0.837, 95% CI: 0.766–0.908, *P* = 0.000).

ROC was calculated for initial degree of luminal stenosis, initial cTFC, postaspiration degree of luminal stenosis, and postaspiration cTFC. Only postaspiration cTFC was predictive of insufficient reperfusion (AUC 0.835, *P* = 0.000) (Figure 2). Initial cTFC (AUC 0.447, *P* = 0.689), initial degree of luminal stenosis (AUC 0.448, *P* = 0.355), and postaspiration degree of luminal stenosis (AUC 0.471, *P* = 0.608) were not correlated to insufficient reperfusion.

### 3.4. Parameters after Aspiration in Patients with Initial Complete Occlusion of IRA

Among patients with initially complete occlusion of IRA, postaspiration cTFC in Group I was higher than that in Group II. There were more patients with postaspiration cTFC > 40 and postaspiration TIMI flow grade 0 in patients with insufficient group (Table 4).

### 3.5. Predictors for Insufficient Reperfusion in Patients with TIMI Thrombus 4~5 Grade

At univariate analysis, hypertension, multivessel disease, time from symptom to PCI (≧4.8 hours), and postaspiration cTFC > 40 were found to be predictive of no-reflow and showed significant *P* values. At multivariate analysis, age ≧ 60 years [OR 6.286, 95% CI 1.477–26.751, *P* = 0.013], hypertension [OR 7.235, 95% CI 1.864–28.076, *P* = 0.004], time from symptom to PCI (≧4.8 hours) [OR 37.926, 95% CI 6.241–230.46, *P* = 0.000], and postaspiration cTFC > 40 [OR 29.273, 95% CI 6.557–130.685, *P* = 0.000] were independently predictive of insufficient reperfusion. Upfront intracoronary GP IIb/IIIa inhibitor administration was an independently positive predictor for sufficient reperfusion [OR 0.044, 95% CI 0.003–0.716, *P* = 0.028] (Table 5).

### 3.6. Predictors for Insufficient Reperfusion in Patients with Initial TIMI Flow Grade 0~1

Among the patients with initial TIMI flow grade 0~1, univariate analysis showed age ≧ 60 years, hypertension, admission GLU, time from symptom to PCI (≧4.8 hours), and postaspiration cTFC > 40 were negative predictors for insufficient reperfusion. At the multivariate analysis, age ≧ 60 years [OR 9.123, 95% CI 1.948–42.710, *P* = 0.005], hypertension [OR 10.762, 95% CI 2.384–48.582, *P* = 0.002], time from symptom to PCI (≧4.8 hours) [OR 43.917, 95% CI 6.554–294.287, *P* = 0.000], and postaspiration cTFC > 40 [OR 27.005, 95% CI 5.757–126.669, *P* = 0.000] were independent predictors for insufficient reperfusion (Table 6).

## 4. Discussions

Early revascularization of infarct-related artery by primary PCI has become the most effective therapy in STEMI. Although PPCI has dramatically reduced the cardiovascular mortality, normal myocardial perfusion is not always restored [16]. Distal embolization of atherothrombotic material during primary percutaneous coronary intervention for STEMI is an important cause of unsuccessful reperfusion [6, 17]. A study showed that distal embolization was associated with a 5-fold increase in 5-year mortality [18].

Embolization of atherothrombotic material downstream in the IRA is related to microvascular obstruction [19]. Manual aspiration catheters are the most commonly used devices because they are easy and safe to use, even in the elderly, and effective manual aspiration of atherothrombotic material before balloon/stent inflation has the potential of decreasing the risks of no-reflow [20]. Previously, single-center randomized clinical trials have shown that manual thrombus aspiration is associated with improved angiographic and electrocardiographic outcomes [9, 21]. However, most recently randomized clinical trials showed limited clinical benefit of routine manual thrombectomy [10–12, 22]. Consequently, recommendation of routine manual thrombectomy was downgraded [23]. The reason why thrombus aspiration results in conflicting data on myocardial reperfusion, infarct size, and clinical outcomes remains incompletely clear. There were few current studies to assess who might benefit most from selective thrombectomy. Detecting clinical predictors for insufficient myocardial reperfusion will help patients with high thrombus burden benefit most from aspiration. In our study, we chose patients with TIMI thrombus grade 4~5 and 97% patients with initial TIMI flow grade 0~1.

According to our study, time from symptom to PCI was longer in patients with insufficient reflow. We took 4.8 hours as a cut-off calculated by ROC curve. Patients with time from symptom to PCI ≧ 4.8 hours may have more frequency of insufficient myocardial reperfusion after PCI. Multivariate analysis showed time from symptom to PCI ≧ 4.8 hours was an independent predictor for insufficient reperfusion. Similar to the study of Yip et al. [24], the no-reflow was lower in those with reperfusion less than 4 hours among patients with AMI who had a high thrombus burden. The underlying mechanism is complex; prolonged ischemia leads to edema of distal capillary beds, swelling of myocardial cells, neutrophil plugging, alterations of capillary integrity, and disruption of microvascular bed, contributing to the pathogenesis of no-reflow [25].

The relationship between reperfusion time and impaired reperfusion could be also illustrated by the study of Kramer and his collogues [26]. According to their study, fresh thrombus (<1 day) is defined as being composed of layered patterns of fibrin and intact platelets, erythrocytes, and granulocytes. Older thrombus (>1 day) consists of lytic (1 to 5 days) and/or organized (>5 days) thrombi. They previously showed that aspirate thrombus fragments may be >12 hours old in STEMI patients with an onset of symptoms of <12 hours. In about half of patients, the thrombus showed features of lytic changes (>24 hours to 5 days) and organization (>5 days). The total ischemia time was significantly longer in patient with older thrombus (4.1 versus 3.3 hours). The longer total ischemic time in the patients with older thrombus may diminish the potential benefit of thrombus removal in these patients [26]. The older erythrocyte-rich thrombi were moderate or large in size. Limited intracoronary material could be retrieved in these patients using thrombectomy devices [27]. With a longer duration to reperfusion, the rigid and older well-organized thrombi tend to fragment with balloon dilatation and may increase the risk of distal embolization during PPCI. Consequently, longer ischemic time associated with older thrombus may be correlated to poor reperfusion and mortality in STEMI treated with aspiration and PPCI. According to TAPSE [9], the trial showed an improved myocardial reperfusion with treatment of aspiration, atherothrombotic material was retrieved in 73% of the patients who underwent thrombus aspiration, and the main constituent of the retrieved material was platelets. In this trial, aspiration was performed soon after the onset of symptoms in a large cohort of patients who were not selected on the basis of angiographic characteristics and were randomly assigned to a treatment group.

In our study, we detected that postaspiration cTFC value was a strong independent predictor for insufficient myocardial reperfusion in patients either with thrombus grade 4~5 or with initial TIMI flow grade 0~1. Furthermore, in patients with initial complete occlusion of IRA, insufficient reperfusion more frequently occurred when postaspiration cTFC values were > 40. This may be due to a variety of mechanisms. Mechanical resistance at the occlusion has a potential role of prevention of passage of the aspiration device through the IRA segment. In some STEMI patients, a high-grade, nonthrombotic, unstable atherosclerotic plaque causes the coronary obstruction [28]. Postaspiration cTFC value in these patients was often >40. In the procedure, some aspiration devices may cause physical damage to the vessel endothelium, which may create new plaque debris, thrombi, or distal embolization, leading to limited effectiveness of aspiration consequently [26].

Inadequate aspiration was a pivotal cause for unsatisfied postaspiration cTFC values. In respect to our study, there were more patients with TIMI flow grade 0 after aspiration in insufficient reperfusion group (16.1% versus 2.9%). In patients with initial complete occlusion of IRA, the percentages were 16.0% and 3.2%, respectively. The older thrombus associated with delayed reperfusion is one of the causes of inadequate aspiration as explained previously. According to our study, more patients with postaspiration cTFC values > 40 had a reperfusion time ≧ 4.8 hours (58.0% versus 42%). The anatomy features of coronary should also be taken into account for inadequate aspiration. Limited intracoronary materials are retrieved in an instance of tortuous vessels. The selection of aspiration device and skills of the operators are pivotal to sufficient aspiration. TIMI flow grade ≧ 2 was a satisfied endpoint. In order to achieve an adequate aspiration, the device has to advance delicately over the thrombotic occlusion to perform continuous intracoronary blood suction. In the case of a large thrombus burden, repeated aspiration or use of a 7 Fr intracoronary manual thrombectomy device or rheolytic tools with greater suction force could be necessary [29, 30].

According to our analysis, patients with hypertension or over 60 years were at great risk for insufficient reperfusion after thrombectomy aspiration treatment. They could be explained by the remodeling of small intramyocardial vessels and interstitial fibrosis, leading to reduced tissue perfusion that easily happened in elderly or patients with hypertension [5]. Our study did not conclude the impact of diabetes mellitus on insufficient reperfusion. According to Iwakura et al. [31], compared to those without no-reflow phenomenon, patients with AMI and no-reflow phenomenon have higher blood glucose levels on admission despite the similar frequency of diabetic mellitus and similar levels of hemoglobin A1C values of the two groups. The study of Ege et al. [32] also suggested that acute hyperglycemia in the acute phase of the MI has relationship with myocardial reperfusion after PCI, probably due to the augmented thrombus formation by hyperglycemia. Univariate analysis in our study showed that admission glucose negatively affected myocardial reperfusion among patients with initial TIMI flow grade 0~1; however, after adjustment for other confounders, the admission glucose was not significantly related to post-PCI cTFC, so a trial in larger sample size is needed in the future to demonstrate the effect of acute hyperglycemia on the myocardial reperfusion after PCI adjunct with thrombectomy aspiration.

Antithrombotic regimen is effective in reducing distal embolization. In a coincidence with the previous studies [33, 34], after multivariate adjustment in our study, upfront intracoronary administration of GP IIb/IIIa inhibitor showed positive effects on myocardial reperfusion after treatment with thrombectomy aspiration, whereas upfront intravenous administration of GP IIb/IIIa inhibitors showed no difference to the reperfusion.

## 5. Limitations

Several limitations of our study should be taken into account. Firstly, the anatomy features of coronary were not described in our study. Secondly our study was not powerful to adjust all the potential confounders, due to small sample size and limited data. Thirdly, elderly or patients with hypertension are susceptive to insufficient reflow; our study was not powerful to evaluate how the advanced age and hypertension impact insufficient reflow in patients treated with thrombectomy aspiration. Fourthly, in a prospective trial, De Vita et al. [35, 36] showed that increasing time to treatment was associated with a significantly decreased reperfusion rate in patients treated with PCI alone. Thrombectomy aspiration limited the adverse effects of increasing time to treatment. Our study only retrospectively observed the clinical predictors for insufficient reperfusion when patients with thrombus burden grade 4~5 were treated with PCI adjunct with thrombectomy aspiration in our center and did not analyze the impact of increasing time on PCI alone versus adjunct with thrombectomy aspiration. Thus, a larger sample, prospective, randomized controlled trial regarding the impact of treatment time on the efficiency of PCI alone versus thrombectomy aspiration is further needed in our further research.

## 6. Conclusion

Age over 60 years, hypertension, time from symptom to PCI (≧4.8 hours), and postaspiration cTFC > 40 were independently predictive of insufficient reperfusion in STEMI patients treated with adjunctive manual aspiration thrombectomy during PCI, whereas early intracoronary GP IIb/IIIa inhibitor Tirofiban administration improved the post-PCI cTFC. The results were consistent in patients with an initial TIMI flow grade 0~1. Identifying these risk factors will improve the effectiveness of PCI adjunct with selective aspiration.

## Figures and Tables

**Table 1:** Baseline characteristics.

Parameters	Group I (*n* = 31)	Group II (*n* = 171)	*P* value
Age (years, $\overset{-}{X}\pm S$)	59 ± 11	56 ± 11	0.108
Age ≧ 60 years	16 (51.6)	57 (33.3)	0.051
Gender, male, *n* (%)	28 (90.3)	149 (87.1)	0.773
Smoker, *n* (%)	18 (58.1)	115 (67.3)	0.321
Hypertension, *n* (%)	24 (77.4)	81 (47.4)	0.002^*∗*^
Diabetes, *n* (%)	10 (32.3)	35 (20.5)	0.147
History of PCI, *n* (%)	1 (3.2)	9 (5.3)	1.000
SBP (mmHg, $\overset{-}{X}\pm S$)	116 ± 18	113 ± 15	0.437
DBP (mmHg, $\overset{-}{X}\pm S$)	73 ± 9	73 ± 10	0.771
Killip classification, *n* (%)			1.000
1~2	2 (6.5)	14 (8.2)	
3~4	29 (93.5)	157 (91.8)	
WBC (×10^9^/L)	11.59 ± 2.77	11.31 ± 3.18	0.650
Red cell count (×10^12^/L)	4.58 ± 0.69	4.64 ± 0.53	0.573
HBG (g/L)	140.41 ± 19.66	143.37 ± 13.90	0.310
Plt-count (×10^9^/L)	193.94 ± 50.30	203.05 ± 51.12	0.361
PDW (%)	12.97 ± 2.52	13.19 ± 2.85	0.736
cTnI (ng/L)	85.93 ± 69.73	89.65 ± 69.64	0.975
ALT (U/L)	60.84 ± 32.33	56.28 ± 41.33	0.310
Scr (*μ*mmol/L)	83.92 ± 24.26	77.51 ± 18.82	0.099
TG (mmol/L)	1.81 ± 0.80	1.65 ± 1.03	0.138
TCHO (mmol/L)	4.68 ± 0.99	4.59 ± 0.97	0.639
HDL-C (mmol/L)	1.04 ± 0.23	0.98 ± 0.22	0.227
LDL-C (mmol/L)	3.01 ± 0.89	3.01 ± 0.88	0.983
Admission GLU (mmol/L)	9.15 ± 4.90	7.87 ± 2.70	0.179

^*∗*^ *P* < 0.01; SBP, systolic blood pressure; DBP, diastolic blood pressure; HGB, hemoglobin, PDW, platelet distribution width; cTnI, cardiac troponin I; ALT, alanine transaminase; Scr, serum creatinine; TG, triglyceride; TCHO, total cholesterol; HDL-C, high-density lipoprotein cholesterol; LDL-C, low-density lipoprotein cholesterol; GLU, glucose.

**Table 2:** Percutaneous intervention findings of patients with insufficient reperfusion and sufficient reperfusion.

Parameters	Group I (*n* = 31)	Group II (*n* = 171)	*P* value
Time from symptom to PCI (hour)	6.2 ± 2.3	4.7 ± 2.3	0.000^*∗*^
Time from symptom to PCI (≧4.8 hours)	26 (83.9)	70 (40.9)	0.000^*∗*^
Initial TIMI flow grade, *n* (%)			0.107
0-1	28 (90.3)	166 (97.1)	
≥2	3 (9.7)	5 (2.9)	
Multiple stents, *n* (%)			0.746
Yes	8 (25.8)	49 (28.7)	
No	23 (74.2)	122 (71.3)	
Multivessel disease, *n* (%)			0.038^†^
Yes	13 (41.9)	41 (24.0)	
No	18 (58.1)	130 (76.0)	
IRA, *n* (%)			0.183
LAD	10 (32.3)	71 (41.5)	
LCX	6 (19.4)	15 (8.8)	
RCA	15 (48.4)	85 (49.7)	
Infarct location, *n* (%)			0.363
Anterior wall	10 (32.3)	70 (40.9)	
Nonanterior wall	21 (67.7)	101 (59.1)	
Initial degree of luminal stenosis (%)	94.60 ± 17.63	98.10 ± 8.58	0.288
Initial cTFC	91.4 ± 22.8	94.3 ± 19.3	0.420
Initial cTFC > 40, *n* (%)	27 (87.1)	160 (94.1)	0.239
Degree of postaspiration luminal stenosis (%)	64.40 ± 22.94	67.66 ± 13.64	0.448
Postaspiration TIMI 0, *n* (%)	5 (16.1)	5 (2.9)	0.009^*∗*^
Postaspiration cTFC	55.5 ± 24.7	30.4 ± 17.0	0.000^*∗*^
Postaspiration cTFC > 40, *n* (%)	21 (67.7)	29 (17.0)	0.000^*∗*^
GP IIb/IIIa inhibitor upfront used			
Intracoronary GPII b/IIIa inhibitor, *n* (%)	2 (6.5)	15 (8.8)	1.000
Intravenous GP IIb/IIIa inhibitor, *n* (%)	4 (12.9)	25 (14.6)	1.000

^*∗*^ *P* < 0.01, ^†^ *P* < 0.05; IRA, infarct-related artery; LAD, left anterior descending; LCX, left circumflex artery; RCA, right coronary artery.

**Table 3:** Correlation between post-PCI cTFC and other parameters.

Parameters	*r*	*P* value
Initial cTFC	0.015	0.832
Postaspiration cTFC	0.569	0.000^*∗*^

^*∗*^ *P* < 0.01.

**Table 4:** Parameters postaspiration in patients with initial complete occlusion of IRA.

Parameters	Insufficient reperfusion (*n* = 25)	Sufficient reperfusion (*n* = 156)	*P* value
Postaspiration cTFC	56.5 ± 24.8	30.5 ± 17.5	0.000^*∗*^
Postaspiration cTFC > 40, *n* (%)	16 (64.0)	27 (17.3)	0.000^*∗*^
Postaspiration TIMI flow grade 0, *n* (%)	4 (16.0)	5 (3.2)	0.022^†^

^*∗*^ *P* < 0.01, ^†^ *P* < 0.05.

**Table 5:** Univariate and multivariate analysis in patients with TIMI thrombus 4~5 grade.

Parameters	Univariate analysis			Stepwise multivariate		
	OR	95% CI	*P*	OR	95% CI	*P*
Age ≧ 60 years	2.133	0.985–4.621	0.055	6.286	1.477–26.751	0.013^†^
Gender	1.378	0.386–4.917	0.621	4.227	0.610–29.289	0.144
Smoking	1.483	0.679–3.241	0.323	0.717	0.185–2.771	0.629
Diabetes	1.850	0.799–4.285	0.151	0.758	0.159–3.614	0.728
Hypertension	3.810	1.558–9.312	0.003^*∗*^	7.235	1.864–28.076	0.004^*∗*^
PCI history	1.667	0.204–13.643	0.634	6.661	0.248–179.053	0.259
Killip class ≧ 3	0.773	0.167–3.585	0.743	0.218	0.020–2.412	0.214
WBC	1.029	0.911–1.161	0.648	1.144	0.940–1.393	0.178
Admission GLU	1.108	0.999–1.229	0.052	1.115	0.889–1.400	0.346
Time from symptom to PCI (≧4.8 hours)	7.503	2.748–20.486	0.000^*∗*^	37.926	6.241–230.46	0.000^*∗*^
Multivessel disease	2.290	1.034–5.071	0.041^†^	2.173	0.493–9.574	0.305
Anterior wall infarction	0.687	0.305–1.548	0.365	1.000	0.308–3.247	1.000
Intracoronary GP IIb/IIIa inhibitor upfront used	0.717	0.156–3.305	0.670	0.044	0.003–0.716	0.028^†^
Intravenous GP IIb/IIIa inhibitor upfront used	0.865	0.279–2.685	0.802	3.359	0.491–22.984	0.217
Initial cTFC > 40	0.422	0.123–1.442	0.169	0.513	0.065–4.034	0.526
Degree of postaspiration luminal stenosis	0.987	0.965–1.010	0.280	0.973	0.935–1.012	0.166
Postaspiration cTFC > 40	10.283	4.384–24.116	0.000^*∗*^	29.273	6.557–130.685	0.000^*∗*^

^*∗*^ *P* < 0.01, ^†^ *P* < 0.05.

**Table 6:** Univariate and multivariate analysis in patients with initial TIMI flow grade 0~1.

Parameters	Univariate analysis			Stepwise multivariate		
	OR	95% CI	*P*	OR	95% CI	*P*
Age ≧ 60 years	2.329	1.036–5.234	0.041^†^	9.123	1.948–42.710	0.005^*∗*^
Gender	1.273	0.354–4.574	0.711	3.940	0.558–27.837	0.169
Smoking	1.306	0.573–2.978	0.526	0.515	0.123–2.146	0.362
Diabetes	1.773	0.738–4.259	0.200	0.613	0.122–3.085	0.552
Hypertension	5.066	1.838–13.964	0.002^*∗*^	10.762	2.384–48.582	0.002^*∗*^
PCI history	1.548	0.188–12.715	0.684	4.732	0.151–148.102	0.376
Killip class ≧ 3	0.835	0.179–3.891	0.819	0.390	0.040–3.761	0.415
WBC	1.031	0.909–1.169	0.638	1.133	0.925–1.388	0.229
Admission GLU	1.123	1.009–1.249	0.033^†^	1.143	0.907–1.441	0.257
Time from symptom to PCI (≧4.8 hours)	6.467	2.343–17.847	0.000^*∗*^	43.917	6.554–294.287	0.000^*∗*^
Multivessel disease	1.973	0.855–4.553	0.111	1.831	0.382–8.770	0.449
Anterior wall infarction	0.666	0.284–1.560	0.349	0.862	0.260–2.862	0.809
Intracoronary GP IIb/IIIa inhibitor upfront used	0.835	0.179–3.891	0.819	0.150	0.013–1.736	0.129
Intravenous GP IIb/IIIa inhibitor upfront used	0.986	0.314–3.094	0.981	3.867	0.529–28.278	0.183
Degree of postaspiration luminal stenosis	0.990	0.966–1.014	0.407	0.973	0.935–1.012	0.166
cTFC > 40 after aspiration	10.405	4.268–23.365	0.000^*∗*^	27.005	5.757–126.669	0.000^*∗*^

^*∗*^ *P* < 0.01; ^†^ *P* < 0.05.
